# Comprehensive characterization of gas dynamic virtual nozzles for x-ray free-electron laser experiments

**DOI:** 10.1063/4.0000262

**Published:** 2024-11-26

**Authors:** Konstantinos Karpos, Sahba Zaare, Dimitra Manatou, Roberto C. Alvarez, Vivek Krishnan, Clint Ottmar, Jodi Gilletti, Aian Pableo, Diandra Doppler, Adil Ansari, Reza Nazari, Alexandra Ros, Richard A. Kirian

**Affiliations:** 1 Department of Physics, Arizona State University, Tempe, Arizona 85287, USA; 2 School of Biological and Health Systems Engineering, Tempe, Arizona 85287, USA; 3 Biodesign Center for Applied Structural Discovery, Arizona State University, Tempe, Arizona 85287, USA; 4 Department of Physics, University of Pennsylvania, Philadelphia, Pennsylvania 19104, USA; 5 Westwood High School, Mesa, Arizona 85201, USA; 6 Maricopa High School, Maricopa, Arizona 85139, USA; 7 School of Molecular Sciences, Arizona State University, Tempe, Arizona 85287, USA; 8 Center for Applied Structural Discovery, The Biodesign Institute, Arizona State University, Tempe, Arizona 85287, USA; 9 School for Engineering of Matter, Transport and Energy, Arizona State University, Tempe, Arizona 85287, USA

## Abstract

We introduce a hardware–software system for rapidly characterizing liquid microjets for x-ray diffraction experiments. An open-source python-based software package allows for programmatic and automated data collection and analysis. We show how jet speed, length, and diameter are influenced by nozzle geometry, gas flow rate, liquid viscosity, and liquid flow rate. We introduce “jet instability” and “jet probability” metrics to help quantify the suitability of a given nozzle for x-ray diffraction experiments. Among our observations were pronounced improvements in jet stability and reliability when using asymmetric needle-tipped nozzles, which allowed for the production of microjects smaller than 250 nm in diameter, traveling faster than 120 m/s.

## I. INTRODUCTION

Gas dynamic virtual nozzles (GDVNs)^1–4^ produce microscopic liquid jets that have been vital for numerous serial femtosecond crystallography (SFX) and time-resolved solution scattering (TR-SS) experiments at x-ray free-electron laser (XFEL) facilities.^5–12^ More than half of the published TR-SS studies and approximately 30% of SFX structures in the Protein Data Bank were produced by using GDVNs.^13^ Although a broad variety of sample injection systems are now available at XFELs,^9^ GDVNs continue to play a key role because they are particularly important for MHz data collection^4,9,11,14–17^ and rapid-mixing experiments.^18–25^

In the context of XFELs, precise control over the microjet characteristics is often paramount. Sufficiently high speeds are critical to ensure that each x-ray pulse interacts with a fresh sample. Jet diameter is consequential for data rates and sample conservation. Stability, perhaps most critical, underpins the reliability of the entire data collection process, as instabilities lead to reduced data rates and can complicate data reduction and interpretation.^26,27^

Poor GDVN performance during XFEL experiments can often be traced back to insufficient pre-testing with specific samples, or a lack of pre-testing altogether. 3D-printed GDVNs^3,4,28,29^ have alleviated much of the variability associated with hand-crafted nozzles,^2,30^ but robust and quantitative pre-testing is still relatively infrequent, especially for groups that do not specialize in GDVN fabrication and development. Various groups have developed methods to quantitatively characterize GDVNs,^3,4,28,31^ but there are presently no open-source, standardized, and comprehensive characterization pipelines that are shared within the XFEL community.

Here, we present a microjet characterization pipeline that consists of a basic hardware configuration and measurement protocols, along with an open-source Python package for data collection and analysis. The pipeline utilizes pairs of frames with short exposures ( ∼100 ns) and applies standard image processing methods to quantify jet speed, length, stability, and diameter. In essence, the pipeline is a specialized variant of particle imaging velocimetry (PIV)^32^ with a graphical interface and analysis procedures tailored to GDVNs and XFEL applications. The results obtained from this pipeline identify the working regions of a GDVN, including drip-to-jet transitions and jet whipping transitions,^33,34^ along with the Weber and Reynolds dimensionless numbers. We demonstrate the utility of the pipeline through quantitative comparisons of microjet characteristics according to nozzle geometric variations along with variations in liquid viscosity. We show how geometric variations can profoundly affect the drip-to-jet transition point and thereby lead to sub-microscopic water jets below 250 nm diameter.^35,36^

## II. METHODS

### A. Experimental setup

**FIG. 1.**
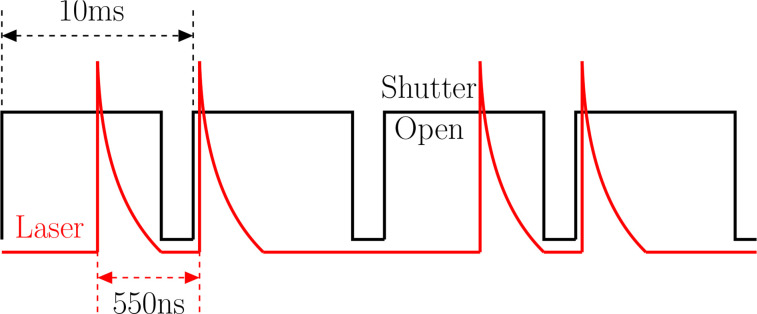
A two-pulse triggering scheme, a technique known as “frame straddling.”

The basic hardware setup for GDVN testing consists of a camera with relatively short readout time (ideally 1 μs or less) along with a double-pulsed laser (ideally 100 ns or less, with pulse fluence of more than 100 nJ/mm^2^). The double-pulsed laser should be timed such that the camera readout occurs during the interval between the laser pulses (a technique known as “frame straddling”), see Fig. 1. The GDVN is controlled by one or more liquid pumps and a gas flow regulator. Ideally, liquid volumetric flow rate (*Q*) and the mass flow rate ( $\dot{m}$) of the gas should be controlled, as these parameters directly map to microjet characteristics without the need to compensate for gas compression or flow resistance in the capillaries. It is helpful to monitor liquid and gas pressures in order to detect leaks or clogs, and it is critical to allow pressures and flow rates to stabilize before recording data.

In the studies presented here, a Photron SA5 high-speed camera was operated at 100 Hz frame rate, with 350 ns readout times. A fiber-coupled diode laser (DILAS D4F4S22 laser with custom pulsed current driver) produced double flashes of 100-ns duration spaced by 550 ns, at 633 nm wavelength. The imaging system consisted of a 10× Mitutoyo long working distance objective paired with a variable Navitar 12× UltraZoom magnifying lens, providing a 3.5 cm working distance. A convex lens was used to focus the diverging output from the 400 μm optical fiber to a spot size of approximately 1 mm diameter. Brightfield images were captured with the light source colinear with the optical axis, whereas quasi-darkfield images were recorded by tilting the axis of the light source and blocking the direct beam.

A Shimadzu (LC-20AD) high pressure liquid chromatography (HPLC) pump was used to drive the liquid flow at specified volumetric rate and to measure the liquid pressure, while Sensirion flow sensors (SLI0430) were used to measure flow rates. To mitigate high pulsation from the HPLC pump, a long glass capillary (40–60 mm in length, with an inner diameter of 100 μm) was included in the setup to provide resistance and dampen pulsations. Before each data collection, the system water was degassed using a sonicator and HPLC purged according to company recommendations to improve stability. Despite these measures, high pulsation, exacerbated by the lack of active feedback in the HPLC pump, remained a significant issue and was the primary source of error in our data collections.

**FIG. 2.**
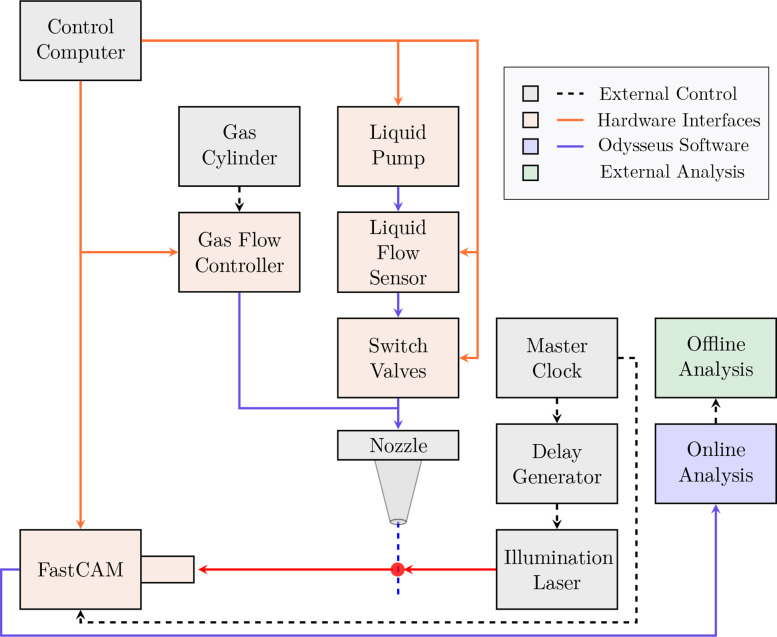
Nozzle testing station flow diagram. Hardware Interfaces and Odysseus software are open-source.

**FIG. 3.**
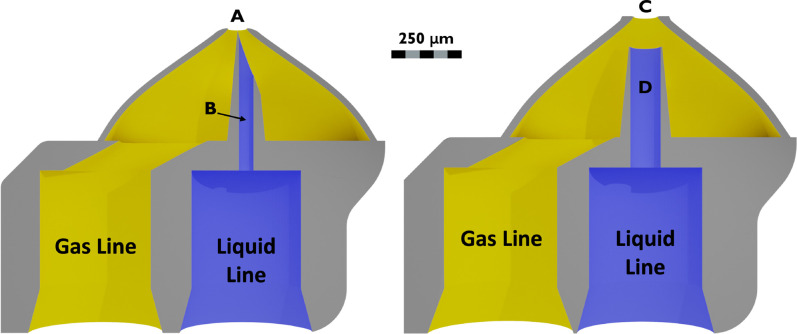
Needle tip (ST) and blunt tip (BT) nozzle sectional views, with gas (yellow) and liquid (blue) lines labeled. (a) 65 μm gas orifice diameter, (b) 50 μm in diameter liquid channel coming to a point in a hypodermic-syringe-type fashion, (c) 80 μm gas orifice diameter, and (d) 100 μm in diameter liquid channel.

A Bronkhorst EL-Flow mass flow controller was used to control and measure the helium mass flow rate. The vacuum chamber was equipped with a rectangular glass chamber and a quick-connect fitting for nozzle mountings, where GDVNs were characterized at vacuum pressures of approximately 0.1 mbar, which was limited by the Edwards XDS35 scroll pump. A hardware schematic is provided in Fig. 2. Nozzle geometries used for data collection are found in Fig. 3.^28^

### B. Custom software

Three specialized software packages have been developed to enable hardware interfacing, microjet image analysis, and a graphical user interface. These open-source Python packages are available for public use.

• hardware_interfaces: This package provides simplified Python interfaces for a range of devices such as HPLC pumps, Photron FASTCam high-speed cameras, and Bronkhorst mass flow controllers, among others.^37^ Client/server classes are based on ZeroMQ, and simple widgets are based on PyQT.
• microjet_analysis: This toolkit is designed for the processing and analysis of GDVN-type jet image stacks, utilizing a custom approach based on particle image velocimetry (PIV) principles. It includes a variety of examples detailing procedures for image enhancement, segmentation, and analysis.^38^ The methods provided in this repository are available in an *online* (data saved in memory) or an *offline* (data save on disk) mode for different types of analysis.
• odysseus: Based upon PyQT and the *pyqtgraph* package,^39^ this package offers a graphical user interface (GUI) that integrates with the *hardware_interfaces* code to manage data collection and control the experimental setup's various facets.^40^ The *microjet_analysis* repository is used in combination with this repository to provide a live, *online*, frame-by-frame analysis for certain jet parameters.

Notably, a significant portion of the software maintains functionality across various operating systems. An exception to this is the code dedicated to the Photron camera, which has a software development kit with pre-compiled libraries that require a Windows operating system (Windows 7 or newer).

### C. Jet image analysis

**FIG. 4.**
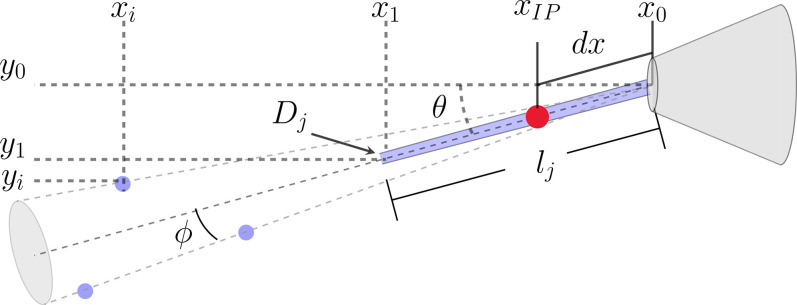
Definitions of key jet characteristics. Here, $l_{j}$ is the jet length, $D_{j}$ is the jet diameter, θ is the angle from a “straight” jet, and ϕ is the droplet dispersion angle. $x_{IP}$ is the “interaction point,” measured a distance *dx* from the nozzle tip. The IP is required for the jet instability measure.

**FIG. 5.**
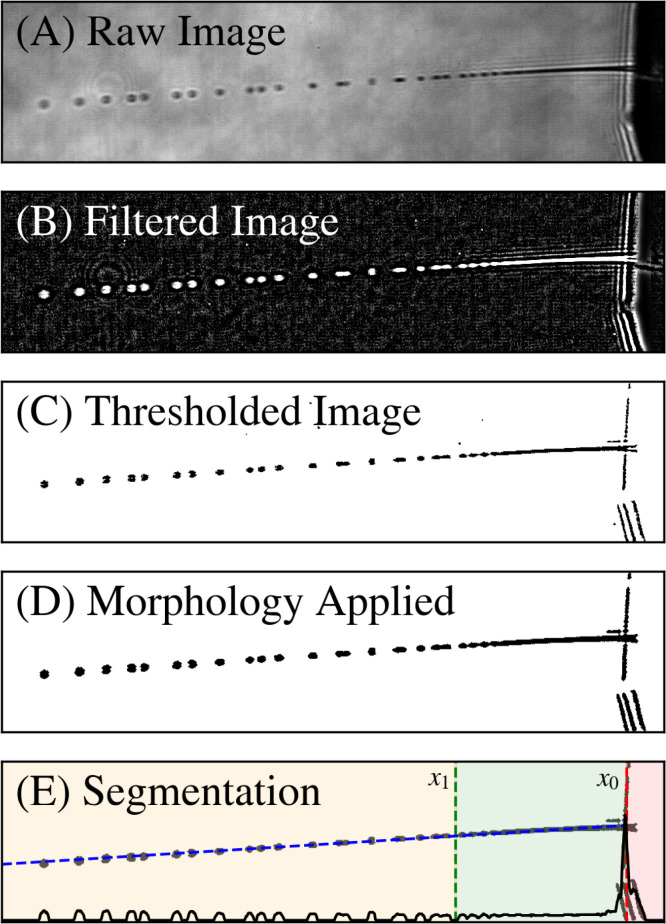
Standard image processing steps with an example dataset. (b)–(d) show the image cleanup steps, while (e) visualizes the segmentation step. In (e), methods are available to automate the nozzle tip ( $x_{0}$, red dashed line) and transition region ( $x_{1}$, green dashed line) index positions. As a simple example of one of the methods, a projected image is displayed in (e) (black solid line), where the individual droplets, continuous jet, and nozzle tip can be easily seen. The blue solid line indicates the jet fit, determined using a Hough transform method for line fitting.

The microjet image processing software employed in this study utilizes image segmentation algorithms to isolate key features such as the nozzle, continuous jet, and droplets, facilitating the statistical analysis of jet attributes including length, instability, and deviation angle, as outlined in Fig. 4. The software calculates average jet speed by analyzing droplet displacements through cross-correlation techniques. While optimizing for a flat and stable background is ideal, the software contains methods to clean images using standard techniques (Fourier filtering, dilation, erosion, thresholding, etc.). The software also allows for both light- and dark-field illumination. The *microjet_analysis* repository contains several practical examples, each illustrating different aspects of image optimization and analysis. Figure 5 delineates the sequential steps involved in refining each frame for subsequent analysis. The methods used to process a single frame are as follows.

#### 1. Image processing

Initially, a high-pass filter is applied to the raw images to remove the non-uniform brightfield background and to enhance the contrast at the edges of the jet and droplets, Fig. 5(b). Subsequent application of hysteresis thresholding, Fig. 5(c), is used to convert images to a binary format. Morphological erosion and dilation operations are then used to eliminate outliers and to fill in voids caused by the high-pass filter in the previous step, Fig. 5(d). These operations leverage standard Python libraries such as *numpy*, *scipy*, and *scikit-image*.

#### 2. Image segmentation

Accurate segmentation of the image into the regions specified in Fig. 4 is necessary for computing parameters such as jet speed and length. Figure 5(e) provides a representation of how processed images facilitate straightforward region determination. The *microjet_analysis* software incorporates several automated procedures for this purpose, with examples illustrating the methods. Here, the 2D jet image is condensed into a 1D projection along the axis perpendicular to the average jet direction, represented by the black line. The peak of this line readily indicates the nozzle tip index ( $x_{0}$). The binary format of the image, consisting of zeros (absence of the jet) and ones (presence of the jet), aids in identifying the transition region ( $x_{1}$), assumed as the starting point of the longest sequence of non-zero elements in the projection, presumed to be the liquid jet.

The Hough Transform is employed to accurately trace the jet's path in an image by pinpointing the most pronounced line, assumed to be along the jet [Fig. 5(e), blue dashed line]. This technique is especially adept at identifying lines even within noisy or discontinuous areas, which are typical in images capturing swift or slender jets.

#### 3. Jet deviation angle, stability, and droplet dispersion

Following from the geometry in Fig. 4, the jet deviation angle is

$$\theta ={\;\text{tan}\;}^{-1}\left(\frac{y_{1}-y_{0}}{x_{1}-x_{0}}\right).$$ (1)

The angular deviation of the *i*th droplet is

$$\phi_{i}={\;\text{tan}\;}^{-1}\left(\frac{y_{i}-y_{0}}{x_{i}-x_{0}}\right)+\theta ,$$ (2)

where the index *i* is the *i*th droplet in the frame. The droplet dispersion is defined as the standard deviation of $\phi_{i}$.

For the purpose of collecting diffraction from a liquid jet, it is important for the jet to be sufficiently stable such that nearly all x-ray pulses intercept the jet. We, therefore, define *jet instability* as the ratio of the fluctuations in lateral jet position to the jet diameter,

$$S_{\text{jet}}=〈\left|dx\;\text{tan}\;\theta \right|〉/D_{j}.$$ (3)

In this equation, *dx* represents the distance from the nozzle tip to the interaction point, $x_{IP}$ in Fig. 4, and is given by $dx=\alpha \;l_{j}$, where α is a dimensionless coefficient ranging from 0 to 1. This coefficient reflects a user-specified percentage of the jet length measured from the nozzle tip, set to 0.5 for all the data presented here. This measurement simulates the typical distance to the point of x-ray interaction, as illustrated in the jet parameter figure. The quotient, thus, provides a quantitative measure of the jet's positional stability in relation to its diameter. Effectively, the larger $S_{\text{jet}}$ is, the more unstable the liquid jet is.

Since the analysis above is applied to 2D projection images of 3D jets, angular deviations from the 3D symmetry axis of the nozzle will be underestimated by a factor of 0.57 on average, and in certain cases, deviations may go undetected. However, XFEL diffraction hit rates are dependent on 2D projections of the target, and thus, the 2D optical characterizations are relevant provided sufficient statistics are gathered.

#### 4. Jet length

**FIG. 6.**
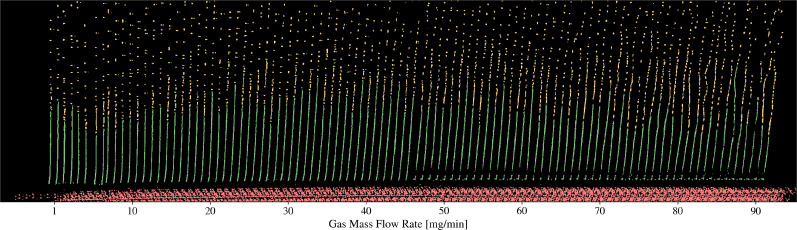
A *birds-eye-view* showing an overlay of many jet images from a dataset with varying gas flow rate. The image stack is sorted by increasing gas flow rate, then translated horizontally by a set distance to give the time-series-style view. When the gas mass flow rate reaches approximately 70 mg/min, the onset of whipping is observed. Jets in this visual were produced with a liquid volumetric flow rate of approximately 5 μl/min. The index positions for the nozzle tip and breakup region were automatically determined using methods from the *microjet_analysis* repository. Breaks in the jet, particularly seen near the nozzle tip in the range 45–90 mg/min, and the missing droplets in the range 34–45 mg/min emphasize the need for optimizing the lighting prior to data collection. The colors reflect the regions discussed in Fig. 5(e).

**FIG. 7.**
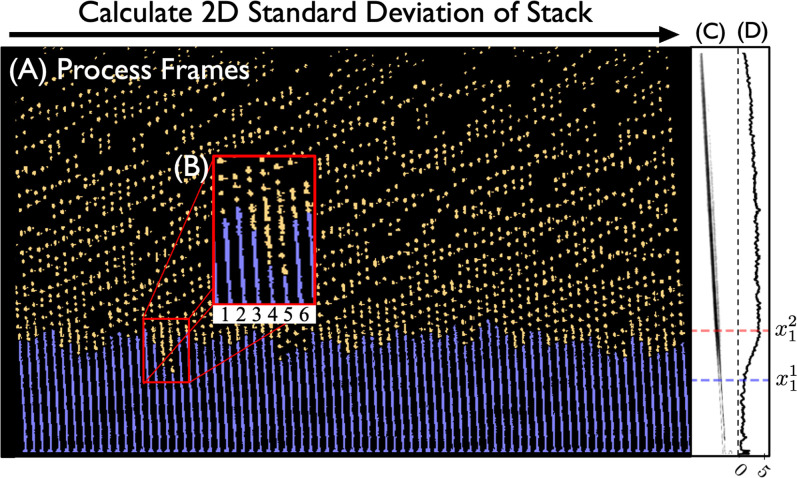
Overview of the *statistical* method to calculating jet length. (a) Birds-eye-view of a single stack of processed images, blue denotes the jet region while yellow denotes the droplet region. The transition between each region is automatically determined using the *connected component* method, used only as a visual in this figure (b) A zoomed in view of the transition regions, with numbers corresponding to the individual frame (jet). (c) The 2D standard deviation of the entire image stack, dark regions represent a high standard deviation. (d) The 1D projected standard deviation, $x_{1}^{1}$ and $x_{1}^{2}$ correspond to the transition index positions between low and high standard deviation, which is indicative of the jet breakup region. The final jet length is calculated by approximating $x_{1}$ from Eq. (4) as the average value between $x_{1}^{1}$ and $x_{1}^{2}$.

Figure 6 presents a *birds-eye-view* of jet images processed using the methods outlined in this paper. While in some cases, the jet length is clearly identifiable by finding the longest segment in the images, there are many cases in which the jets are far smaller than the resolution of our microscope ( ∼ 2–3 μm), and therefore, jet length estimates are more difficult. We, therefore, introduce two methods for estimating jet lengths. The first method identifies the largest connected component in a processed image [see Fig. 5(d)]. The second method calculates the standard deviation across a stack of frames to estimate the breakup point, shown in Fig. 7. These are referred to as the *connected component* and *statistical* methods, respectively.

The *connected component* method defines the jet length as

$$l_{j}=\frac{\left|x_{1}-x_{0}\right|}{\;\text{cos}\;\theta },$$ (4)

where $x_{1}$, $x_{2}$, and θ are defined according to Fig. 4. This method tends to overestimate jet lengths in cases where droplets cannot be resolved, as shown in the inset of Fig. 7(b).

The *statistical* method provides a more accurate estimate in cases of sub-micrometer jets or poorly focused images. In this case, the jet length is defined by detecting fluctuations in the jet images, which are present despite the fact that individual droplets cannot be identified.

We quantify these fluctuations by computing the 2D standard deviation of the image stack, as shown in Fig. 7(c). A 1D projection of this shows a sigmoidal-like function, as shown in Fig. 7(d). The breakup region is indicated as the region between $x_{1}^{1}$ and $x_{1}^{2}$. We define the jet length as the average of these two points.

The *statistical* method can be automated by utilizing a soft-edge-finding algorithm (e.g., a 1D peak search applied to a smoothed derivative of the 1D profile is effective), but this algorithm is not yet formally implemented in our analysis software package. Future updates to the *microjet_analysis* repository will introduce more robust methods for jet length determination. However, accurate measurement still depends heavily on optimal lighting and imaging systems.

#### 5. Jet probability—A measure of a nozzle's drip-to-jet transition

Our study introduces jet probability as a metric, denoting the fraction of images that exhibit a jet. This measure is derived from an analysis of segmented regions downstream of the nozzle tip. The presence of a jet is discerned by identifying the longest stretch of connected pixels within the image, implying a continuous jet. Each image is assigned a Boolean value to reflect this presence or absence. These Boolean values are subsequently averaged in bins according to liquid and gas flow rates, as depicted in Fig. 12. Multiple methods are available in the *microjet_analysis* repository for this metric, all of which use a minimum pixel length to account for image artifacts or small pixels. Values below this threshold are understood as no stable jet. This technique helps identify nozzles that produce spurting jets that are unreliable for x-ray diffraction measurements.

#### 6. Jet speed and diameter

Since jet diameters are often too small to resolve with imaging, we infer diameters from jet speed and liquid flow rate measurements. Average jet speeds are measured by averaging cross correlations between pairs of images separated by a small time delay. We employ two types of cross correlations.^41^ The first is an ordinary 2D cross correlation averaged over many image pairs:

$$C_{ij}=\sum_{n}\sum_{kl}A_{kl}^{2n}A_{k+i,l+j}^{2n+1},$$ (5)

where $A_{ij}^{n}$ is the *n*th binary image in a sequence and $A_{ij}^{n+1}$ is the *n*th time-delayed binary image captured after a delay of Δt=550 ns following $A_{ij}^{n}$. The images contain only the droplet regions, which results in a sharp peak in $C_{ij}$ that corresponds to the average displacement vector $\vec{s}$. The jet speed is $U_{j}=|\vec{s}|d_{\text{pixel}}/\Delta t$, where $d_{\text{pixel}}$ is the size a pixel (assumed square). Assuming that the droplet speed matches the jet speed near the breakup region, the jet diameter is

$$D_{j}=2\sqrt{\frac{Q}{\pi U_{j}}}.$$ (6)

The second type of cross correlation is the average outer product,

$$P_{nk}=\sum_{i=1}^{n}a_{2i,k}\;a_{2i+1,n}-a_{2i+1,k}\;a_{2i+2,n},$$ (7)

where for n=1,2,…,N, *a* is an N×K matrix, with *n* representing the number of frames and *k* the number of data points. Here, *a* represents the projections of $A_{ij}^{n}$ and $A_{ij}^{n+1}$ onto the line that best fits the jet.

Figures 22 and 23 show typical correlations $C_{ij}$ and $P_{nk}$. While the correlation $C_{ij}$ tends to be most reliable, the correlation $P_{nk}$ is helpful because it allows for the determination of droplet speeds as a function of distance from the nozzle and hence enables the detection of droplet acceleration. For jets produced in vacuum, droplet acceleration is usually negligible, and hence, the droplet speeds determined from $C_{ij}$ are sufficient.

Although it is possible to track individual droplets and infer the speeds of each of them, such an approach is challenging for high-speed jets (which require very short delays Δt) and sub-micrometer drops (which are difficult to detect reliably). It is also possible to compute 2D cross correlations in localized regions of each image pair, which would yield a velocity vector field akin to common PIV methods. In our experience, we find that the $C_{ij}$ and $P_{nk}$ are sufficiently robust for GDVN microjet analysis.

#### 7. Outlier filtering

Our data collection employs two main outlier filtering methods to ensure data quality. The first method addresses individual frame anomalies in image stacks, removing any frames where the mean pixel intensity of an individual frame exceeds the mean pixel intensity of the entirety of the stack. The second filtering approach involves analyzing jet speed. It assesses the projection of correlation peaks (detailed in Appendix B). Datasets are discarded if the peak correlation cannot be distinguished from background correlations, based on a predefined threshold. This method is crucial for identifying and excluding datasets with unreliable or ambiguous jet speed measurements. Both methods are detailed in the *microjet_analysis* repository examples.

### D. Drip-to-jet transition data collection method

GDVN microjets exhibit hysteresis whereby the boundary between jetting and dripping modes depends on the history of the gas and liquid flow rates. With a fixed gas flow rate $\dot{m}$, the liquid drip-to-jet transition point $Q_{DJ}(\dot{m})$ can be found by increasing the liquid flow rate until a stable jet is established. Once the jet is established, the liquid flow rate can be reduced to the jet-to-drip transition point $Q_{JD}(\dot{m})$ where the jetting mode switches back to the dripping mode. Here, the notation $Q(\dot{m})$ represents the liquid jet as a function of the gas mass flow rate, $\dot{m}$. The transition points $Q_{DJ}(\dot{m})$ and $Q_{JD}(Q\dot{m})$ depend somewhat on the rate at which the liquid flow rate is changed. A jet that is established in the region $Q_{JD}<Q\leq Q_{DJ}$ is quasi-stable in the sense that a small disruption can cause a switch to dripping, at which point the jet is unlikely to be established until the liquid flow rate is increased. Similar transition points can be found by holding the liquid flow rate fixed while varying the gas flow rate.

Since our intention is to determine the properties of *stable* jets, we seek the drip-to-jet transition points $Q_{DJ}(\dot{m})$ in order to avoid quasi-stable states. Data are collected at fixed gas flow rate, and the initial liquid flow rate is reduced until the dripping mode is established. The liquid flow rate is then slowly increased until the jet is reestablished. In general, we avoid reducing the gas flow rate to the corresponding jet-to-drip transition point ${\dot{m}}_{JD}(Q)$ because low gas flows often result in liquid wetting the nozzle and freezing due to evaporative cooling in the low-pressure environment, which can cause nozzles to fracture.

**FIG. 8.**
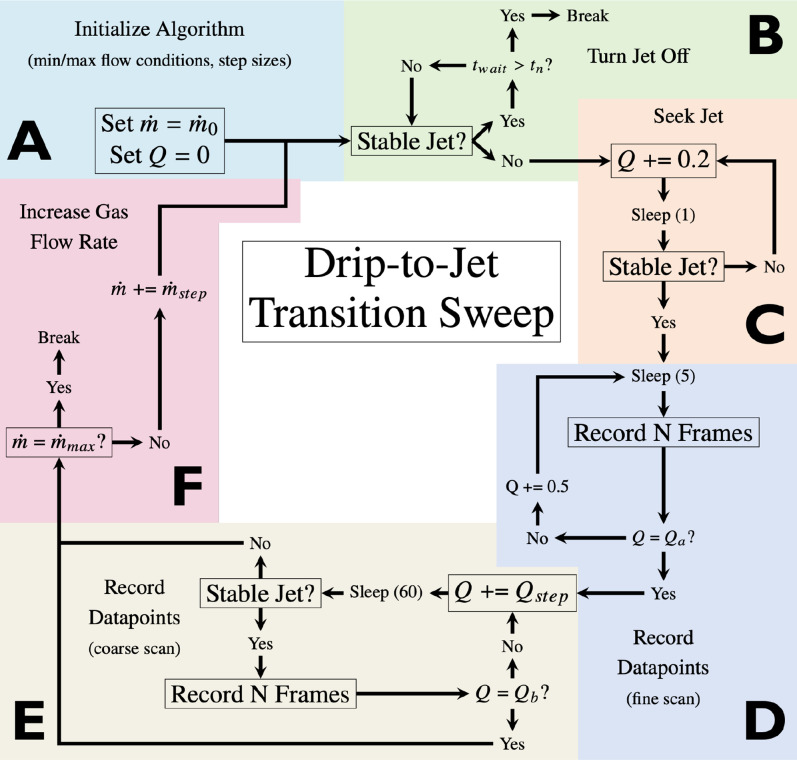
Flow chart for the drip-to-jet transition sweeping algorithm. (a) Initialization, setting $\dot{m}$ to a user-defined gas mass flow rate with no liquid flow. (b) Waiting for the jet to stop, where $t_{n}$ is a user-defined waiting parameter, and $t_{\text{wait}}$ is the time spent waiting for the jet to stop. (c) Slowly increase *Q* until a stable jet is found. (d) Record a stack of images, increase the flow rate up 0.5 μL/min and record until a user-defined maximum is achieved ( $Q_{a}$). The purpose of this step is to finely sample the drip-to-jet transition region. (e) Coarsely sample Q to a maximum of $Q_{b}$. (f) Increase $\dot{m}$ by a user-defined step size ${\dot{m}}_{\text{step}}$ and restart the algorithm.

Figure 8 schematically illustrates the data collection algorithm. The process starts at an initial gas mass flow rate ( $m_{0}$), incrementing up to a predetermined maximum ( ${\dot{m}}_{\text{max}}$), with the size of each increment given by ${\dot{m}}_{\text{step}}$. After the detection of the transition point, the liquid flow rate is modified in steps of $Q_{\text{step}}$ until it reaches a specified maximum rate ( $Q_{b}$). This procedure can be automated, with the drip-to-jet transition determined by image analysis procedures detailed previously to calculate the percentage of recent frames that exhibit a stable jet, discussed in Sec. II C 5). The “Sleep” function mandates a waiting period after each flow rate adjustment, allowing the system to reach equilibrium and ensuring that the flow rates measured upstream of the nozzle accurately reflect the flow rates at the nozzle tip.

## III. RESULTS

### A. Jet characterization across different nozzles

**FIG. 9.**
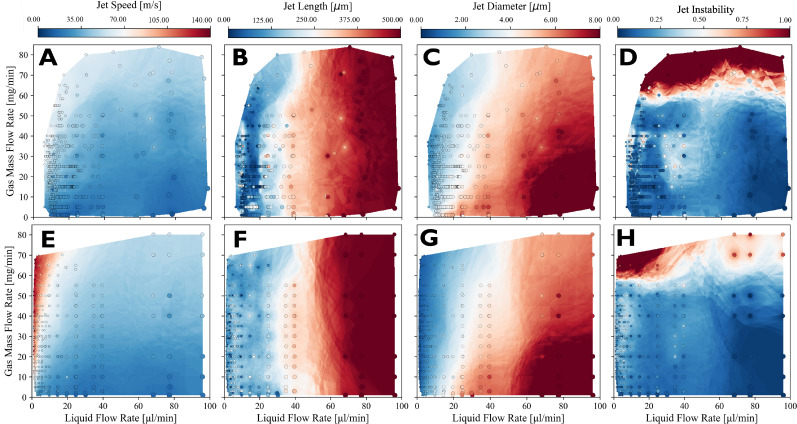
Side-by-side comparison of jet speed (a) and (e), jet length (b) and (f), jet diameter (c) and (g), and jet instability (d) and (h) for blunt tip (a)–(d) and needle tip (e)–(h) GDVNs running water. Data are aggregated from four different nozzles for each type. Each circle corresponds to a stack of individual images (between 120 and 250 frames). Circle colors indicate average values while diameters are scaled in proportion to average jet diameters. The leftmost datapoints correspond to the drip-to-jet transitions. The colormap filling the region between datapoints is calculated using the k-nearest neighbors algorithm. For jet lengths greater than 450 μm, the droplet region exceeded the field of view of the camera such that the nozzle tip had to be moved out of frame. Due to this, the jet length was noted to be greater than 450 μm, but the exact length is unknown. All jet lengths in this figure were calculated using the *connected components* method.

Figure 9 shows a comparison of jet speed, length, diameter, and stability across needle-tip and blunt-tip nozzle geometries, as a function of both liquid (water) and gas (helium) flow rates. The aggregated data, derived from four distinct nozzles per geometry, adhere to the experimental protocol outlined in Fig. 8 so that the lowest liquid flow rates correspond to the drip-to-jet transition points. The results show a pronounced advantage of needle-tip nozzles in facilitating jet formation at significantly lower liquid flow rates compared to blunt-tip nozzles. As a result, needle-tip nozzles are capable of generating jets that are much smaller and faster than the blunt-tip geometry. This distinctive performance of needle-tip nozzles can be attributed to their specialized geometry, seen in Fig. 3, which promotes the formation of a narrow liquid thread that remains adhered to the solid substrate. The resulting Couette-like flow field effectively mitigates instabilities induced by recirculation patterns.^42^

**FIG. 10.**
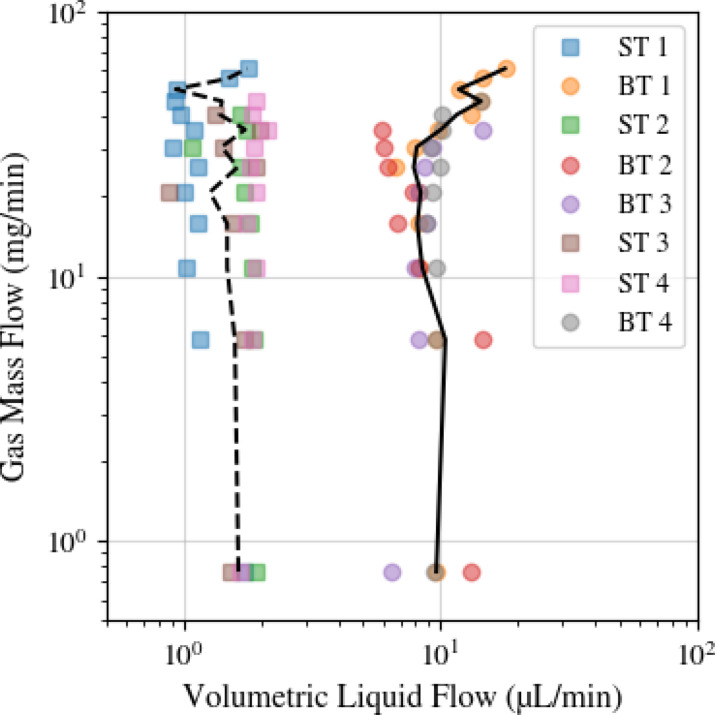
Variation in nozzle print job. Each uniquely colored point represents the drip-to-jet transitions of a unique nozzle. Square markers indicate needle-type (ST) nozzles, while circular markers indicate blunt tip (BT) nozzles. The transition regions were determined by binning $\dot{m}$ for each unique nozzle, identifying the lowest *Q* within each bin. The black lines were determined by calculating the average transition point across nozzles for each bin.

Figure 10 highlights the differences in drip-to-jet transition points among different print jobs of the same nozzle geometry, indicating to the significance of geometric precision in nozzle manufacturing for consistent performance. This variation suggests that even minor differences in nozzle geometry can critically affect jet formation and stability.^43^ The figure demonstrates that the nozzle designs generally remain within 3 σ of the mean value. This variability is likely dominated by geometric variation but may be partly attributable to liquid flow rate errors (approximately 1%–10%).

**FIG. 11.**
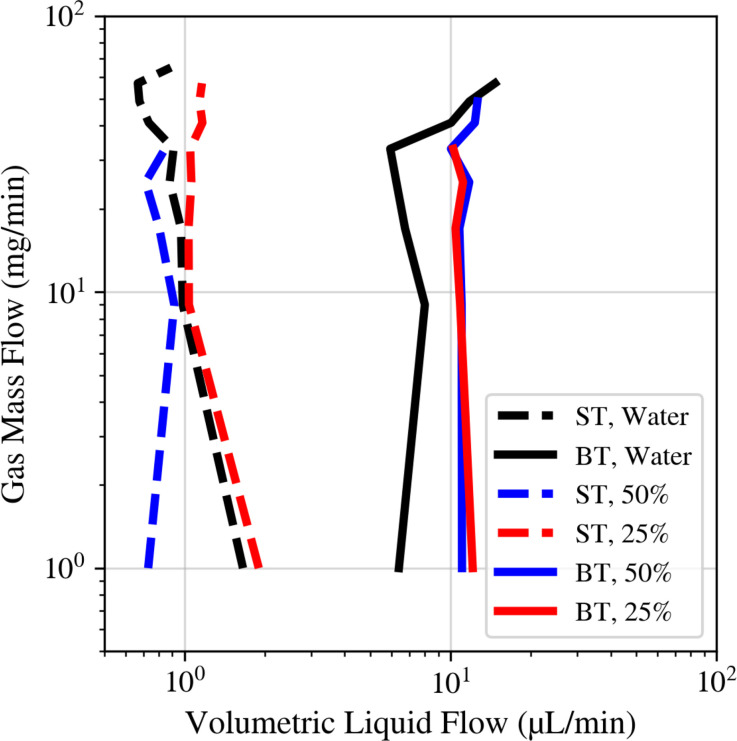
Drip to jet transitions for the lowest achievable *Q* across a large $\dot{m}$ range. Black coloring represents water, red represents a 25% v/v water/glycerol mixture, and blue indicates a 50% v/v water/glycerol mixture. Dashed lines and solid lines represent the needle (ST) and blunt tip (BT) nozzles, respectively. The transition regions were found by binning $\dot{m}$ for each nozzle/sample-type combination, determining the lowest *Q* within that bin, and fitting a line to the corresponding data points. Chemical properties for each mixture can be found in Table I.

Figure 11 examines how water–glycerol mixtures influence drip-to-jet transitions, revealing an absence of a consistent trend with fluid viscosity and surface tension changes. This finding indicates that nozzle geometry and operational conditions may overshadow the fluid's properties in determining jet behavior at the drip-to-jet transition region.

**FIG. 12.**
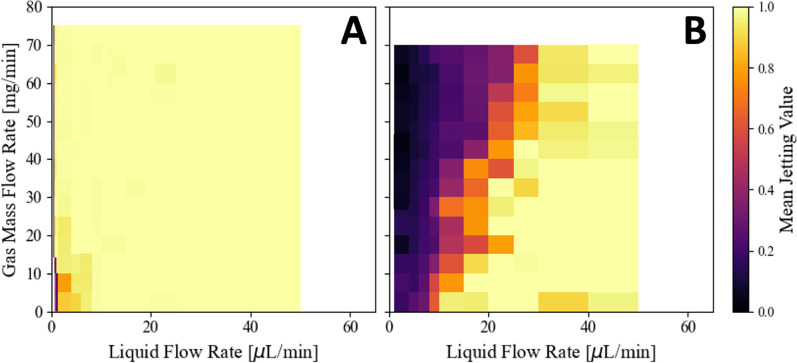
A measure of a nozzle's ability to form a stable jet for a needle tip (a) and blunt tip (b) nozzle. Data collection followed the methods discussed in Secs. II D and II C 5. Lack of data at certain flow rates is indicated by white coloring.

Figure 12 shows the fraction of images that contain jets, providing a statistical measure of the drip-to-jet transition. This transition often includes a spurting phase before stabilizing into a consistent jetting phase. For each type of nozzle, only one was used in the experiments due to the extensive effort required for data collection. Notably, all blunt tip nozzles demonstrated a similar spurting region, and the observed drip-to-jet transition regions correspond closely with those depicted in Fig. 10. Note the liquid flow rate scales are different in each figure.

Our analysis reveals distinct behaviors between the two nozzle designs. Needle-tip nozzles consistently maintain jet stability across a broad range of flow rates. Conversely, blunt tip nozzles show a gradual increase in their ability to form stable jets, with performance dropping significantly—entering a “spurting” phase—below an 80% jet probability score. This phase is characterized by the inability to maintain a stable jet, indicating less effective performance under certain conditions. These observations, derived from the methodology specified in Sec. II C 5, provide clear evidence of the differences in performance between needle and blunt tip nozzles.

### B. Probing the Limits of GDVNs

**FIG. 13.**
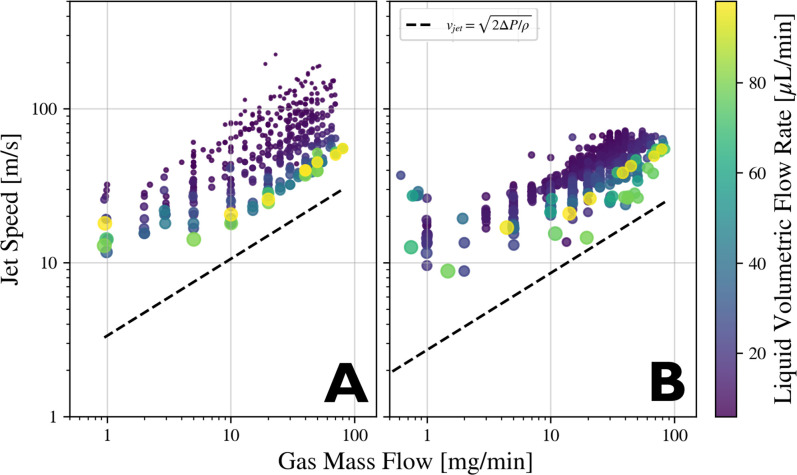
Scatter plots of gas mass flow rate vs measured jet speed. Colormap indicates liquid volumetric flow rate. Marker size is proportional to jet diameter. Sample is water. Black dashed lines indicate the predicted jet speeds according to the Bernoulli equation $U_{j}=\sqrt{2\Delta P/\rho }$. Details on measuring the internal nozzle pressure, ΔP can be found in Appendix A. Figures (a) and (b) use a needle and blunt tip nozzle, respectively.

**FIG. 14.**
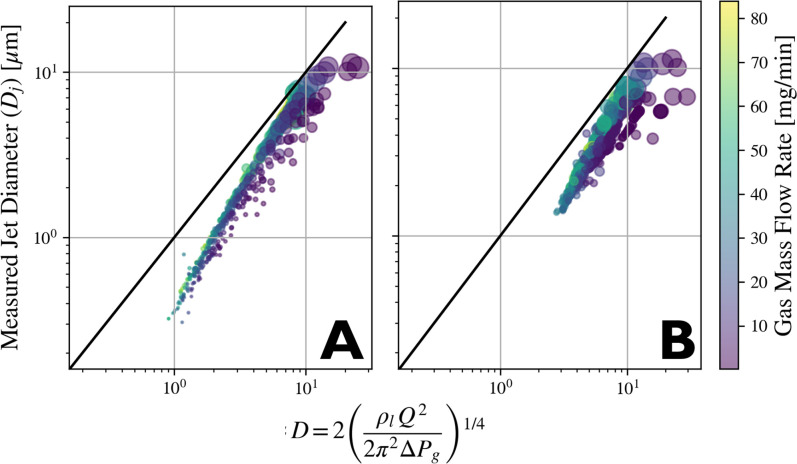
Scatter plots of jet diameters as predicted by the 2011 paper by Gañon-Calvo *et al* and the experimentally determined values.^44^ Colormap indicates gas mass flow rate. Marker size is proportional to *Q*. Sample is water. Black line indicates the one-to-one line between the x- and y-axes to serve as a guide to the eye. Figures (a) and (b) use a needle and blunt tip nozzle, respectively.

Figure 13 compares jet speeds to the predictions based on the Bernoulli equation $U_{j}=\sqrt{2\Delta P/\rho }$, while Fig. 14 compares the measured $D_{j}$ with those calculated using the equation $D_{j}\approx D=2{\left(\frac{\rho_{l}\;Q^{2}}{2\pi^{2}\Delta P}\right)}^{1/4}$, which neglects the effects of surface tension and viscosity. Here, ΔP is calculated using the methods discussed in Subsection 1 of Appendix. Observed jet speeds consistently exceed those predicted by the Bernoulli equation, while jet diameters are consistently smaller, highlighting the significant role of viscous drag in influencing jet characteristics. The discrepancy between measured values and the predictions lessens with increased liquid flow rates, as would be expected due to the reduced ratio of surface area to volume. Differences in nozzle design notably influence these observations. We hypothesize that needle-tip nozzles tend to be significantly faster and smaller than the Bernoulli equation predicts because the geometry promotes longer durations in which drag forces can accelerate the fluid before it emits from the tip as a free-standing liquid that is subject to Rayleigh breakup.

**FIG. 15.**
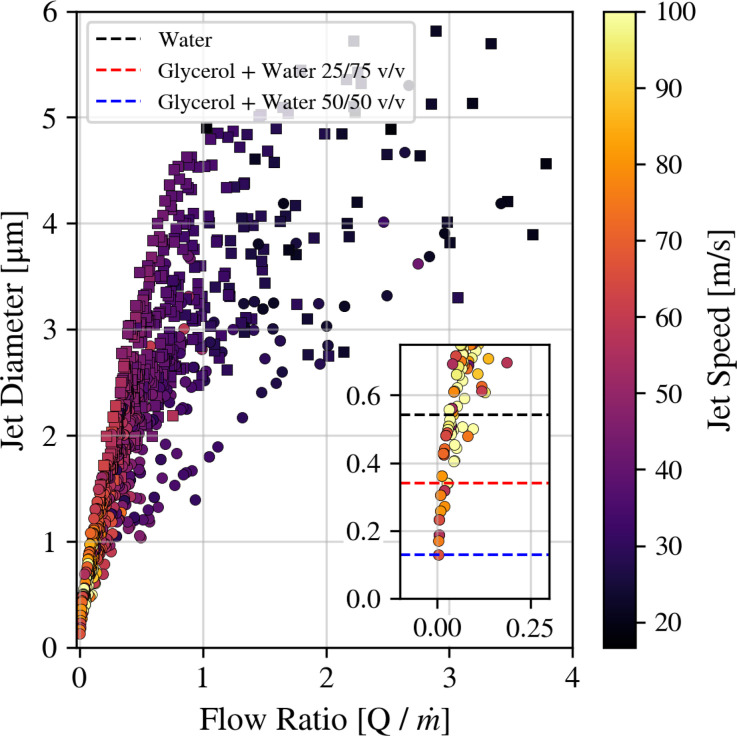
Scatter plot of flow ratio vs jet diameter. Square markers indicate the blunt tip nozzle, while circular markers represent the needle tip. The color bar indicates jet speed. The plot combines 3627 unique data points across the same samples and nozzle designs seen in Fig. 11. An inset plot is included, focusing on the range from 0 to 0.75 μm jet diameter, highlighting our ability to measure jets below 200 nm in diameter. The dashed lines indicate the lowest measured diameter for each sample type.

**FIG. 16.**
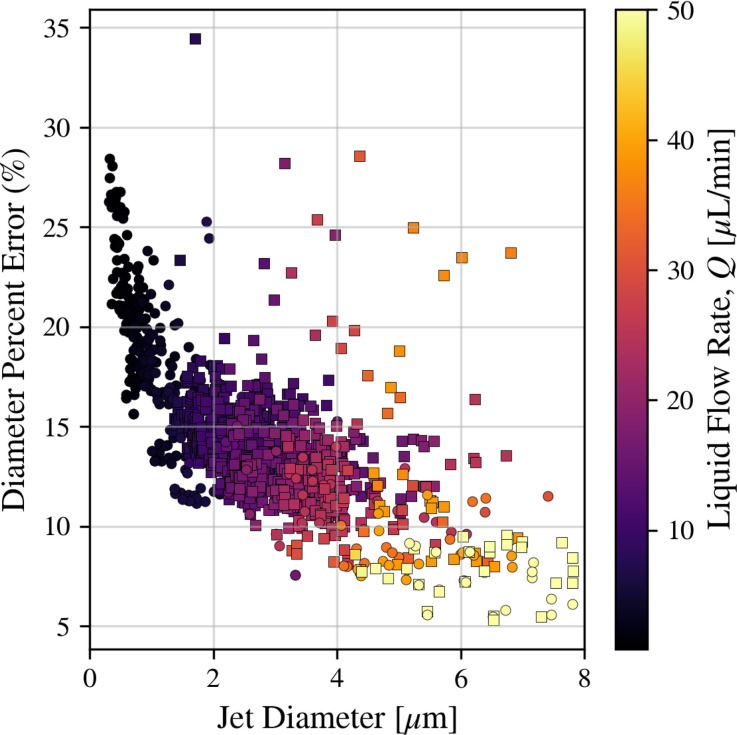
Scatter plot of the jet diameter percent errors. As *Q* decreases, and subsequently $D_{j}$ decreases, the relative error associated with the HPLC pump increases significantly. Square markers indicate the blunt tip nozzle, while circular markers represent the needle tip. The color bar indicates the volumetric liquid flow rate, *Q.*

Figure 15 shows jet diameters as a function of flow ratio $Q/\dot{m}$, with data from experiments using both needle- and blunt-tip nozzles and fluids including water and water/glycerol mixtures. Lower flow ratios tend to correspond to smaller, faster jets due to the reduced liquid mass and the increased viscous forces due to the gas. As shown in the inset of Figure 15, sub-micrometer jets are frequently observed, with the smallest jets approaching 100 nm diameter. Notably, jet diameters below 250 nm are predominantly associated with the needle tip geometry. Jet diameter errors are dominated by variations in the HPLC pump. As we reach the lower limits of the HPLC, the jet diameter errors are greater than 20%, as seen in Fig. 16. The inset figure also indicates that the lowest measured diameter is dependent on the sample type. As glycerol concentration increases, the smallest achievable jet decreases, presumably due to the reduction in capillary wave frequencies associated with increased viscosities.

### C. Dimensionless numbers

The dimensionless numbers are defined below, where $U_{j}$ is the jet velocity, $D_{j}$ is the jet diameter, ρ is the liquid density, σ is the surface tension, and μ is the liquid dynamic viscosity. Table I provides relevant numerical values.

**TABLE I.** Liquids used and some of their physical properties^1^ at 24.5 °C. Glycerol is slightly hygroscopic, which may lead to minor variations in the actual concentrations of the glycerol–water mixtures.

Liquid	ρ (kg/m^3^)	σ (N/m)	μ (cP)
Water	997	0.0728	0.89
Water + Glycerol 75/25 v/v	1078	0.067	2.76
Water + Glycerol 50/50 v/v	1030	0.066	6.17

The liquid jet Reynolds number (Re) compares inertial forces to viscous forces within the jet through the ratio

$$\text{Re}\equiv \frac{\rho \;U_{j}D_{j}}{\mu }.$$ (8)

Reynolds numbers higher than ∼ 1500 typically correspond to turbulent flows.^28,45,46^ As seen in Fig. 18(a), all of the liquid jet Reynolds numbers in our datasets are well under this limit, suggesting that laminar flows within the jets are likely.

The sheath gas Reynolds number (Re_*g*_) compares inertial forces to viscous forces at the nozzle exit through the ratio

$${\text{Re}}_{g}\equiv \frac{\rho_{g}U_{g}(d_{g}-D_{j})}{\mu_{g}}=\frac{4{\dot{m}}_{g}}{\pi \mu_{g}(d_{g}-D_{j})},$$ (9)

where $d_{g}$ is the gas orifice diameter (65  μm for the needle-tip and 80 μm for the blunt-tip nozzles according to Fig. 3), and $\dot{m}=\rho_{g}\;U_{g}\;\pi \;{\left(d_{g}-D_{j}\right)}^{2}/4$ is the measured gas mass flow rate. For the calculations here, $\mu_{g}=1.96\times 10^{-5}$ Pa s.^47^

**FIG. 17.**
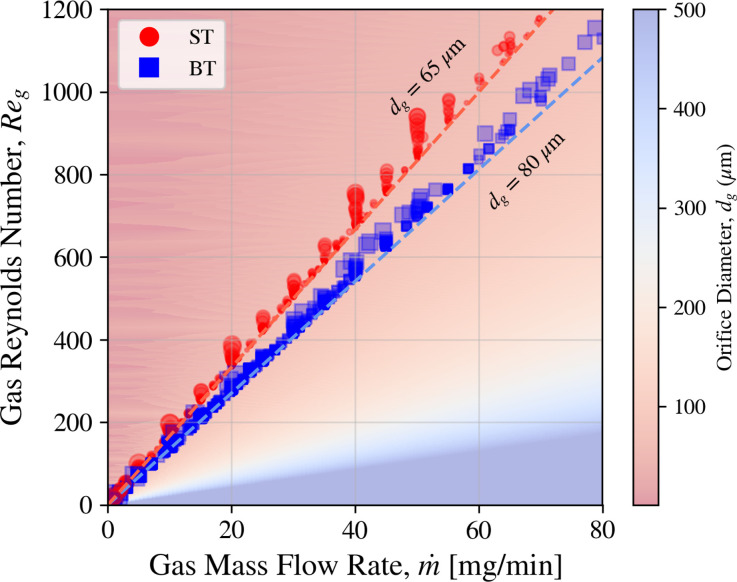
$Re_{g}$ vs $\dot{m}$ for blunt (BT) and needle tip (ST) nozzles as calculated from Eq. (9), marker size is proportional to $D_{j}$. Sample is water. The background gradient shows the result of Eq. (9) with $D_{j}=0$ for various gas orifice diameters. Dashed lines highlight the values of Re_*g*_ with $D_{j}=0$ for the needle and blunt tip nozzle designs.

**FIG. 18.**
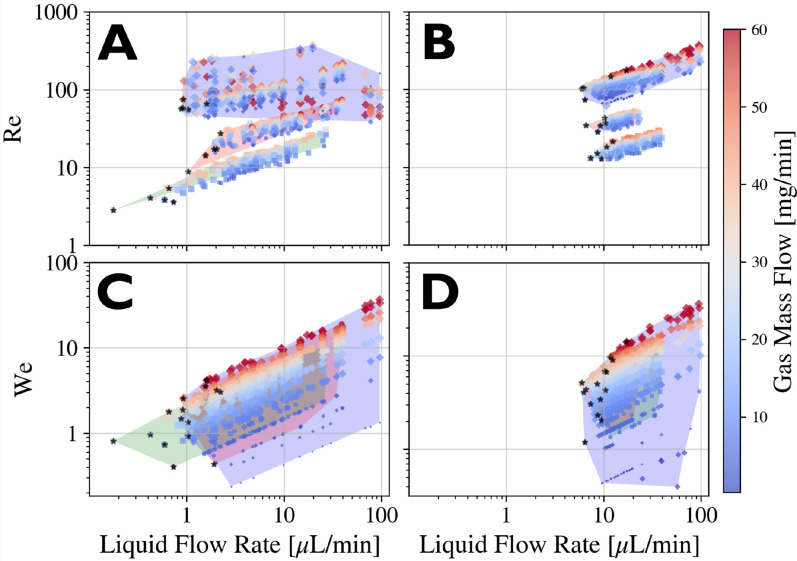
Combined Weber and Reynolds numbers vs liquid volumetric flow rate with water (blue background, diamond markers), 75/25 water/glycerol (red background, circle markers), and 50/50 water/glycerol (green background, square markers). Marker size is proportional to $D_{j}$. The color bar indicates the gas mass flow rate. Black stars indicate the lowest achievable liquid flow for each gas range recorded, essentially lining out the drip-to-jet transition region. Figures (a) and (c) were collected with needle tip nozzles, while figures (b) and (d) were collected with a blunt tip nozzle.

Figure 17 shows Re_*g*_ according to each nozzle geometry, using the $\dot{m}$ data discussed throughout. As expected, the blunt-tip geometry has a propensity for higher Re_*g*_ due to the smaller gas orifice diameter. This observation suggests that faster and smaller jets can be achieved by decreasing $d_{g}$, which should be tested in future studies. While the liquid jet Re is safely under 1500 [Fig. 18], Re_*g*_ surpasses values of 900 which suggests an onset of gas turbulence for both nozzle designs.

The liquid jet Weber number (We) compares inertial forces against surface tension through the ratio

$$\text{We}\equiv \frac{\rho \;U_{j}^{2}D_{j}}{2\sigma }.$$ (10)

In order for a stable liquid jet to form, inertial forces must be overcome surface tension forces (We >1). From Figs. 18(c) and 18(d), we see that the typical drip-to-jet transitions occur in the range 1≲We≲80. An obvious trend in $\dot{m}$ is observed; as $\dot{m}$ increases, $U_{j}$ increases and subsequently the numerical value for the dimensionless number also increases. As seen in Figs. 9(d) and 9(h), high $\dot{m}$ is associated with whipping instabilities. This suggests a characteristic We range for stable jets.

**FIG. 19.**
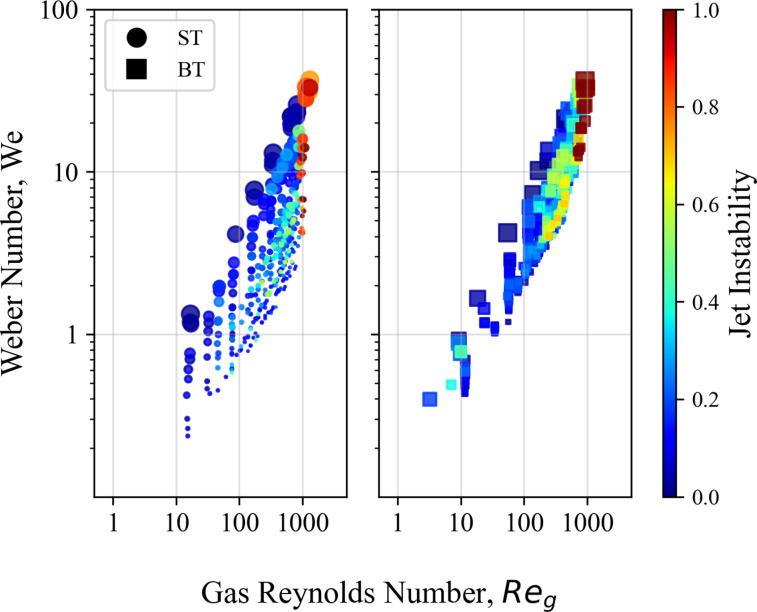
$Re_{g}$ vs We for blunt and needle tip nozzles, marker size is proportional to *Q*. Sample is water. Jet instability values of 0 imply a perfectly stable jet and 0.5 a moderately stable jet. Values greater than 0.9 imply a highly unstable jet, according to the definition in Eq. (3).

GDVNs have two control knobs: $\dot{m}$ and *Q*. The Weber number describes the conditions of the liquid jet, while the gas Reynolds number offers insight into the dynamics of the helium gas sheath surrounding the liquid jet. Figure 19 plots these two values, highlighting their effect on jet stability. The blunt-tip nozzles show a region of instability where the liquid flow rates are small compared to the gas mass flow rates, as indicated by marker size and values of Re_*g*_ according to Eq. (9). The jet instability begins at Reg≈250, which corresponds to a $\dot{m}$ of approximately 25 mg/min. This is further corroborated by the result in Fig. 12, where Q<20 μl/min produce highly unstable jets at this gas mass flow rate. Figure 19 indicates that the blunt-tip nozzles operate best in regions where both We and Re_*g*_ numbers are low. A similar trend with the needle-tip nozzles is observed, although the instability begins when *Q* is very low with respect to $\dot{m}$, perhaps due to the nozzles geometry.

A 2021 article by Gañan-Calvo *et al*^48,49^ theorized that the jet length follows the below equation:

$$\;\frac{L_{j}\;\Delta P}{\sigma }=\alpha_{\rho }\;\zeta ,\;\zeta ={\text{We}}^{2}{\left[{\left(\text{We}+\alpha_{\mu }^{2}{\text{Ca}}^{2}\right)}^{1/2}-\alpha_{\mu }\text{Ca}\right]}^{-1},$$ (11)

where $\alpha_{\rho }$ and $\alpha_{\mu }$ are dimensionless constants and Ca is the capillary number defined as^50,51^

$$\text{Ca}=\frac{\mu U_{j}}{\sigma }.$$ (12)

**FIG. 20.**
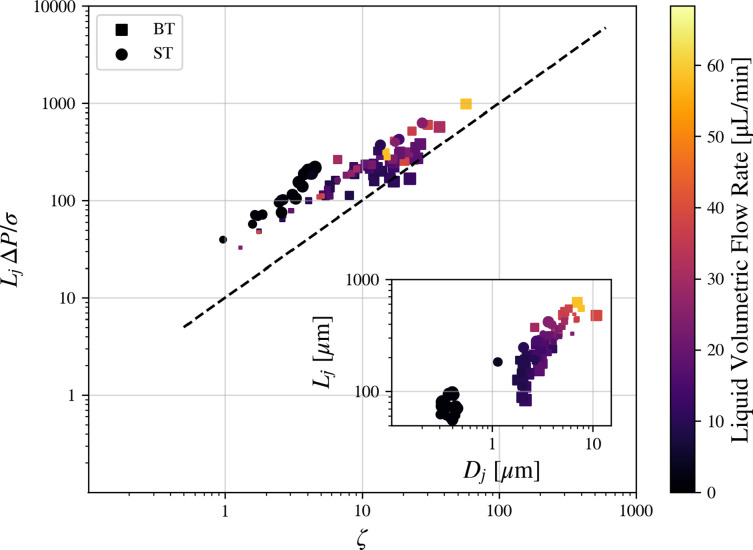
Jet length $L_{j}$ as a function of ζ, as defined in Eq. (11) (dashed line). Color map represents the liquid flow rate, while marker size is proportional to $\dot{m}$. Marker style differentiates between the blunt and needle tip nozzles according to the legend.

**FIG. 22.**
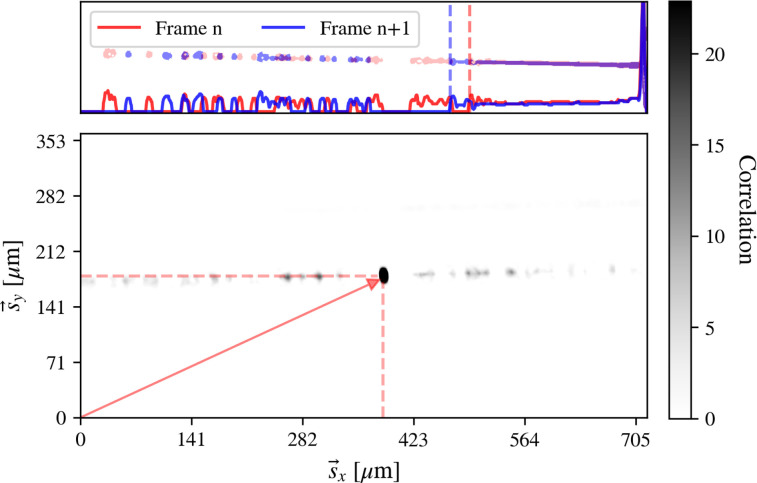
A typical 2D cross correlation, $C_{ij}$. The top figure shows two sequential jet images, with dashed lines corresponding to the jet breakup region. The cross correlation calculation only uses the data downstream of the dashed lines.

**FIG. 23.**
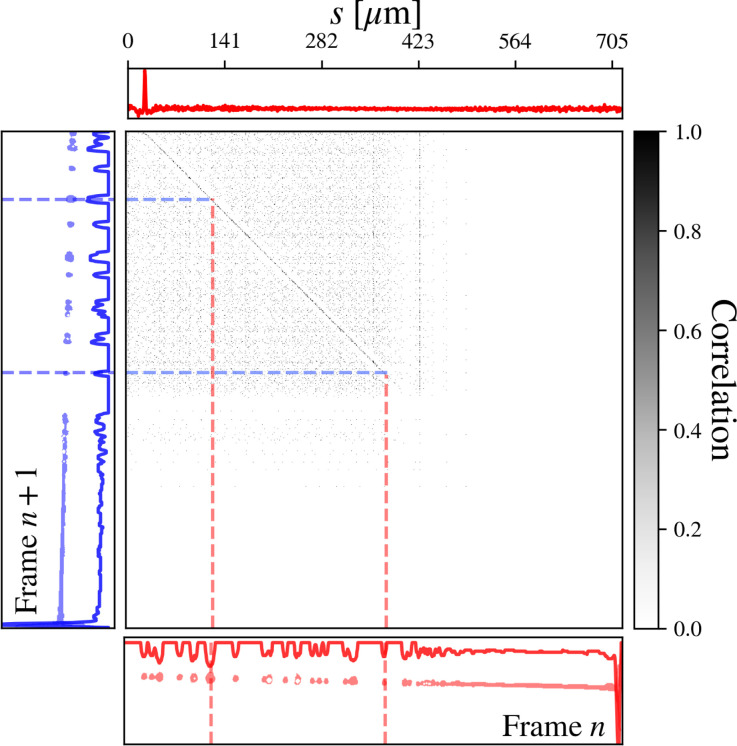
A typical outer product correlation matrix, $P_{nk}$. Two sequential frames are displayed to the left and below the correlation matrix, with dashed lines indicating two chosen droplets. The correlation matrix, displayed in the middle, shows the output of $P_{nk}$ in Eq. (7). The distance from the white zero-line to the correlation maximum (bright line just north of the white line) gives a droplet displacement according to Eq. (A2). The plot north of the correlation matrix shows the projection of sheared matrix. The distance from s=0 to the peak (seen here at approximately s=35) provided an average droplet displacement.

Figure 20 shows remarkable agreement with the findings of Gañan-Calvo *et al.*, further validating the model proposed by the authors.

## IV. CONCLUSION

The primary objective of this study was to demonstrate a hardware-software system for comprehensive characterization of liquid microjets used in XFEL experiments. The system combines hardware with open-source Python software to automate data collection and analysis, addressing a critical need for standardized and reproducible characterization methods in the XFEL community.

Key findings of this study include the successful demonstration of the system's ability to measure and analyze essential jet characteristics such as speed, length, diameter, and stability. Comparative results between needle-tip and blunt-tip nozzle geometries revealed the superior performance of needle-tip nozzles, which produced smaller, faster, and more stable jets. Notably, the system achieved the production of microjets smaller than 250 nm in diameter, traveling at speeds exceeding 120 m/s.

Liquid Reynolds numbers were generally below 200, suggesting laminar conditions, while liquid Weber numbers correlated with the transition from dripping to stable jetting, indicating a characteristic range of stability ( 1≲We≲80). Gas Reynolds numbers had values that likely surpassed 900 in some cases, suggesting the onset of gas turbulence at higher gas mass flow rates. High gas Reynolds numbers correlated with high degrees of jet instability, suggesting the onset of gas turbulence as a possible mechanism for jet whipping behavior.

We have introduced the jet probability metric, defined as the fraction of images exhibiting a continuous jet, to assess the reliability of nozzles for XFEL measurements. Plots of jet probability revealed that blunt-tip nozzles exhibited a remarkably gradual transition from dripping to jetting as liquid flow rates were increased, which obscures the boundary between different operational modes such as “jetting,” “dripping,” and “spurting.” No mechanism for these gradual transitions were identified.

Three samples were tested: water, 25% glycerol–water, and 50% glycerol–water mixtures. The results showed a clear trend in which increased viscosity allowed for the formation of smaller jets. However, the results also showed that drip-to-jet transitions varied between nozzle print jobs to a greater extent than they varied between different liquid viscosities.

While we generally observed that needle-tip nozzles consistently outperformed blunt-tip nozzles by multiple metrics, we did not assess how these metrics are affected by the addition of particles in the solution, such as protein crystals. Without further studies, we caution against concluding that needle-tip nozzles are superior to blunt-tip nozzles for SFX experiments, especially in the case of large microcrystals. However, our results strongly suggest that needle-tip nozzles are superior for solution-scattering studies.

The largest source of errors in our measurements were the variations in liquid flow rates, which were often at the 10% level due to the mechanical action of the HPLC liquid pumps that were used. Variations in print quality also played a clear and significant role in quantifying nozzle performance. The study observed that even slight differences from one print job to the next could impact jet characteristics, with the jet-to-drip transition and jet instability metrics being affected most strongly. The tendency of blunt-tip nozzles to enter an extended “spurting” phase suggests a need for further investigation to determine whether this problem is caused by the design or the quality of the print jobs. In addition to improving liquid flow rate stability and printing quality, other high-priority improvements to the presented system would be the use of an incoherent nanosecond light source to minimize coherent speckle, and a camera with much higher data transfer rates.

In summary, we successfully demonstrated a hardware–software system capable of characterizing liquid microjets for XFEL experiments. The software is open-source and available for others to use. Routine application of such a system could greatly reduce the likelihood of costly sample-delivery surprises during diffraction data collection. Rapid jet characterization with high accuracy can be highly impactful to the validation of computational fluid dynamics simulations and nozzle geometry investigations, which can further expose important fluid physics and geometrical factors needed to perfect the liquid microjet nozzle.

## Figures and Tables

**FIG. 21.**
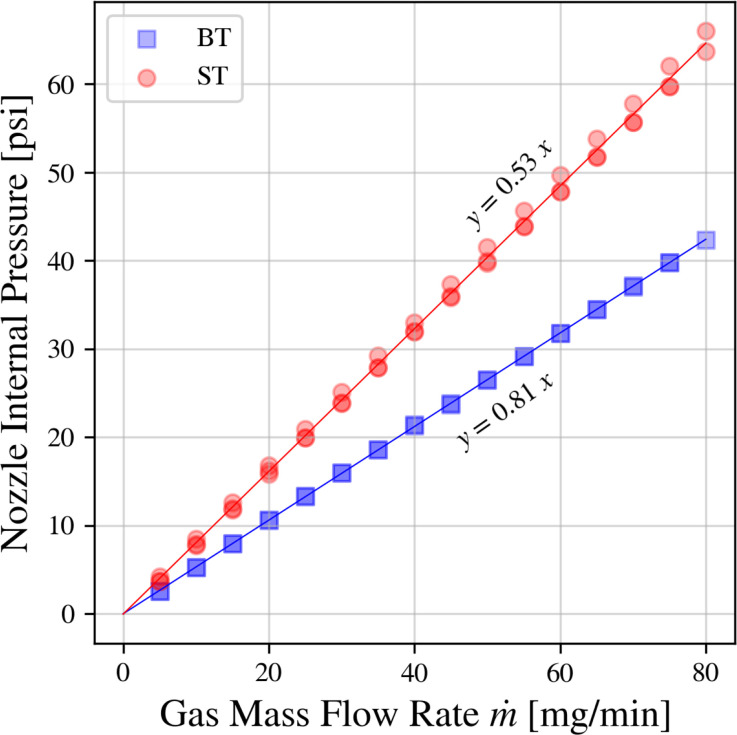
Internal nozzle pressure mapping. Red circles represent the needle-tip nozzle, while blue squares represent the blunt tip. Two nozzles from each design were tested. Helium gas used for all tests, which were done in a vacuum environment.

## Data Availability

The data that support the findings of this study are available from the corresponding authors upon reasonable request.

## APPENDIX A: ESTIMATION OF THE INTERNAL NOZZLE PRESSURE

Accurately determining the pressure drop ( ΔP) across the GDVN gas orifice is necessary for some comparisons of data against theory. The standard 100- μm ID gas capillaries create significant flow resistance, and therefore, the inlet pressure is a poor measure of the internal nozzle pressure. We, therefore, determined the relationship between measured gas mass flow rates and the internal nozzle pressures by printing GDVN variants with the liquid lines plugged and the bases modified to accept large 500- μm-ID gas capillaries. We confirmed that this eliminated all flow resistance by fixing the mass flow rate and checking that the capillary length did not affect the inlet pressure (i.e., the gas orifice was the only significant source of flow resistance). Internal pressures were recorded as a function of mass flow rate for two print jobs of each of the two nozzle geometries. The external pressure was 0.1 mbar. Thorough leak checks were conducted, and pressure readings were cross-verified with two independent pressure gauges. The results of these measurements are shown in Fig. 21. It is clear that the internal nozzle pressure has a nearly ideal linear relation with the mass flow rate, which is expected for choked flow. Linear fits to the data ( $P=\alpha \dot{m}$) yielded coefficients of α=0.53 psi/mg/min for the blunt-tip GDVNs, and α=0.81 psi/mg/min for the needle-tip GDVNs, which are close to the values we predicted (0.46 and 0.73 psi/mg/min, respectively) based on the choked-flow assumption.

## APPENDIX B: JET SPEED BY CORRELATIONS

Figure 22 shows a typical 2D cross correlation defined by Eq. (5). In this case, the displacement vector, $\vec{s}$, is shown as the vector from the i=0, j=0 coordinate to the correlation maximum. To calculate the average displacement,

$$\left|\vec{s}\right|=\sqrt{{\left({\vec{s}}_{x}\right)}^{2}+{\left({\vec{s}}_{y}\right)}^{2}},$$
(A1)

where ${\vec{s}}_{x}$ and ${\vec{s}}_{y}$ are the *x*- and *y*-components of $\vec{s}$. The calculated jet speed using this method, for the data presented in the figure, is $U_{j}=44.88$ m/s.

The same dataset is applied to the outer product calculation, described in Eq. (7) and shown in Fig. 23. To calculate the average droplet displacement from Eq. (7), we perform a shearing operation as

$${\tilde{P}}_{nk}=P_{n,(n+k)\text{mod}(n)}$$
(A2)

to shift the correlation maximum. Once shifted, we calculate the average droplet displacement as

$$s=\text{argmax}\left(\sum_{i=1}^{n}{\tilde{P}}_{ik}\right).$$
(A3)

For the dataset presented in Fig. 23, the calculated jet speed is $U_{j}=44.89$ m/s.
